# An overlooked source of skin dose perturbation: Commercial tattoo inks in radiotherapy

**DOI:** 10.1371/journal.pone.0346501

**Published:** 2026-04-02

**Authors:** Hongjun Park, Beechui Koo, Jungwook Shin, Byoung Hyuck Kim, James J. Sohn

**Affiliations:** 1 Department of Chemical and Biological Engineering, Northwestern University, Evanston, Illinois, United States of America; 2 Department of Radiation and Cellular Oncology, The University of Chicago, Chicago, Illinois, United States of America; 3 Data Science Institute, The University of Chicago, Chicago, Illinois, United States of America; 4 Division of Cancer Epidemiology and Genetics, National Cancer Institute, National Institutes of Health, Rockville, Maryland, United States of America; 5 Department of Radiation Oncology, Seoul National University College of Medicine, Seoul, Republic of Korea; University of Malaya, MALAYSIA

## Abstract

Approximately one-third of US adults have tattoos, yet the dosimetric impact of intradermal tattoo pigments during radiation therapy remains uncharacterized. Commercial tattoo inks contain unregulated metallic impurities including chromium, lead, and nickel, raising concerns about dose perturbations in tattooed skin. This work quantifies radiation dose perturbations induced by high-atomic-number (Z) tattoo pigments under clinically relevant radiotherapy conditions. Monte Carlo simulations (TOPAS) modeled layered skin phantoms with a 0.3-mm intradermal tattoo layer embedded at 1.25–1.55 mm depth. Three commercial inks were evaluated: carbon-based (black) and metal-containing (Fe-rich brown, Al-containing orange) at pigment loadings of 5–100 vol% within the tattoo layer, to establish upper-bound effects. Electron (6, 18 MeV) and photon (6, 18 MV) beams were simulated with standard clinical geometry (1 × 1 cm² field, SSD = 100 cm). Photon irradiation produced pronounced, depth-localized dose enhancement, with peak dose enhancement factor (DEF) reaching 2.5 for brown ink at 18 MV, a 62% mean increase relative to non-tattooed skin driven by high-Z–mediated secondary electron production. Electron beams exhibited energy-dependent behavior: 6 MeV produced modest enhancement (peak DEF ~ 1.07), while 18 MeV unexpectedly generated dose deficits (DEF < 1.0) due to enhanced lateral scattering. Critically, all perturbations remained depth-confined without lateral propagation, preserving spatial dose uniformity across tattooed and non-tattooed regions. Tattoo pigments containing toxic metals create substantial localized dose enhancements under photon irradiation but minimal perturbations under electron therapy. These modality-dependent effects represent a previously unrecognized source of dose uncertainty in radiotherapy and warrant consideration in treatment planning for the growing population of tattooed patients.

## Introduction

Tattooing has become a common, permanent form of body art. Population surveys indicate that 32% of adults in the United States and a growing proportion worldwide have at least one tattoo [1]. This widespread practice intersects with the central role of radiation therapy in cancer care. Projections for 2025 and beyond indicate that more than half of all cancer patients expected to receive radiation during the course of treatment [2,3]. As the prevalence of tattoos increases, the question of whether intradermal tattoo pigments could measurably alter skin dose has direct relevance for patient counseling, treatment planning, and regulatory guidance.

Commercially available tattoo inks are heterogeneous mixture of pigments, dispersants, and additives [4,5]. Black inks are typically carbon based, whereas colored inks often contain metal oxides (e.g., iron oxides), other organic (e.g., azo dyes) or organometallic compounds (e.g., copper phthalocyanine) [5,6]. Multiple analyses of commercially available tattoo inks have reported variable levels of toxic metals such as Ni, Cr, Cd, and Pb, with some products exceeding recommended limits under regional frameworks such as the European Union REACH restrictions [7–9]. By contrast, tattoo inks fall under cosmetic regulation by the U.S. FDA; no color additives are explicitly approved for intradermal injection (i.e., tattooing), and oversight focuses primarily on microbiological safety rather than toxicological and radiological properties [10–12]. These observations motivate a physics-based assessment of whether metal-containing components, particularly those with a higher atomic number (Z), could perturb therapeutic dose deposition in the skin.

Analogous concerns have previously arisen in other imaging and treatment settings. Early case reports of heating and burns in tattooed patients undergoing magnetic resonance imaging (MRI) led to significant attention, but subsequent systematic investigations demonstrated that clinically relevant effects are rare when safety guidelines are followed [13,14]. A similar, quantitative evaluation is warranted for therapeutic photon and electron beams [15]. Dosimetric commissioning and reference measurements (e.g., AAPM TG-106) ensure accuracy under standard conditions [16], and local dose perturbations from implants and tissue heterogeneity are well documented [17,18]. Because tattoos are dermally embedded, often within treatment fields [19], and can contain high-Z components, they may modify superficial dose. However, existing data on tattoo-radiation interactions are yet insufficiently characterized or represented in current clinical, toxicological, and regulatory frameworks [20–22].

In this context, a key unmet need is a modality-resolved analysis of how realistic tattoo pigment scenarios influence skin dose under clinically relevant external beam radiotherapy (EBRT) geometries. Herein, we address these gaps by quantifying skin dose perturbations from tattoo pigments using Monte Carlo simulations with anatomically informed skin models that incorporate a sub-millimeter intradermal tattoo layer.

We construct pigment composition scenarios ranging from carbon-based (black color) to metal-containing (brown and orange) inks. Monoenergetic 6 and 18 MeV electron and 6 and 18 MV photon beams are examined at a standard source-to-surface distance (SSD) of 100 cm. For each configuration, depth-dose distributions were computed within a 2.4-mm skin slab, and resulting perturbations were analyzed as a function of pigment concentration and anatomical location. By demonstrating upper bounds on dose changes and identifying conditions under which localized hotspots arise, this work aims to inform clinical decision-making for tattooed patients undergoing EBRT and to provide a quantitative framework for subsequent experimental and clinical validation.

## Materials and methods

### Ink composition

Tattoo pigments were represented using three commercial Intenze tattoo inks selected based on a published market survey [4]. The inks included a brown and orange ink containing elevated Pb and Cd levels, and a black ink with negligible heavy metal content. Elemental compositions originally reported on a wet-mass basis (including solvent) [4] were converted to a dry-mass basis to represent the solid pigment fraction retained in the dermal tissue. A representative dry-matter content of 47 wt% (typical range 31–62 wt%) was adopted from the Danish Environmental Protection Agency survey (2012) [23].

The dry-mass elemental concentrations were directly implemented into TOPAS material definitions. Component densities were assigned according to their realistic chemical forms rather than elemental state: Fe_2_O_3_ (5.3 g/cm^3^), used as a representative iron oxide pigment commonly reported in commercial brown tattoo inks [4,23], Al(OH)_3_ (2.4 g/cm^3^) additives for the orange ink, and carbon black (1.8 g/cm^3^) for the black ink. Mixtures in TOPAS were defined by weighing each elemental component according to its reported mass fraction. This approach produces physically accurate, chemically representative densities and avoids overestimation that would result from using pure elemental densities. The resulting elemental mass fractions and compound-based densities were then used to generate tattoo-dermis mixture materials at 0, 5, 10, 25, 50, 75, 100 vol% pigment loadings, which served as direct material inputs for tool for particle simulation (TOPAS) simulations.

### Skin layer design

**Fig 1:**
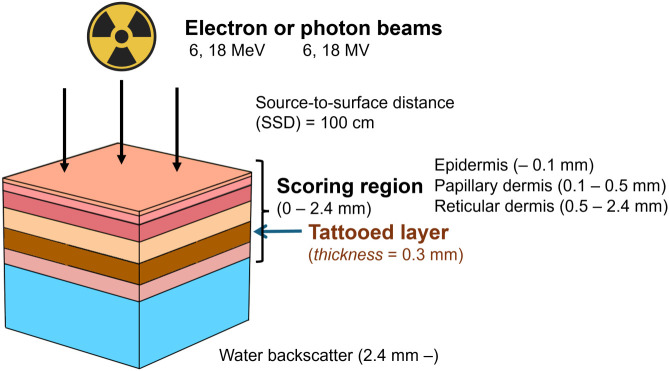
Schematic illustration of the irradiation setup used in the Monte Carlo simulations.

Although realistic tattoo pigment concentrations are typically below 25 vol%, higher loadings (up to 100 vol%) therefore represent parametric upper-bound scenarios rather than biological concentrations and systematically characterize concentration-dependent effects. To investigate these dosimetric perturbations, a voxel-based multilayer skin model was implemented in the TOPAS, as illustrated in Fig 1. The geometry reproduced major anatomical skin structures, including the epidermis, papillary dermis, and reticular dermis, using literature-based thicknesses, densities, and compositions [24,25].

The simulation geometry consists of a multilayer skin model comprising the epidermis and dermis sublayers with a 0.3-mm tattooed layer was embedded within the reticular dermis at depths of 1.25–1.55 mm below the skin surface. The lateral simulation area (1 × 1 cm^2^) was used, or divided into four quadrants containing brown, orange, black tattoo pigments, and a control region without ink. Irradiation was performed using electron or photon beams with an SSD of 100 cm. Energy deposition was scored in the 0–2.4 mm skin region, and a water phantom beneath the skin layer was included to a model backscatter condition.

The epidermis was modeled as a 0.1-mm layer consisting of six sublayers: the upper stratum corneum (upper SC, 8.5 µm, 1.3 g/cm^3^), middle SC (12.8 µm, 1.2 g/cm^3^), lower SC (12.8 µm, 1.1 g/cm^3^), stratum granulosum (8 µm, 1.1 g/cm^3^), stratum spinosum (50 µm, 1.1 g/cm^3^), stratum basale (8 µm, 1.1 g/cm^3^). Beneath the epidermis, the 2.4-mm dermal region (2.4 mm total) consisted of a 0.4-mm papillary dermis (1.1 g/cm^3^) overlying a 1.9-mm reticular dermis (1.1 g/cm^3^).

We derived elemental compositions for each skin layer from published sublayer analyses [24,26], consisting predominantly of oxygen (62 wt%), carbon (20%), hydrogen (10%), and nitrogen (3%). The remaining ~ 5 wt% consisted of physiologically relevant trace elements, including sodium and potassium (~ 1.4% each), phosphorus (1.6%), sulfur (0.4%), and minor quantities of calcium (0.07%), zinc (0.013%), and iron (0.019%). These average compositions were applied uniformly across the skin layers for material definition in TOPAS.

The tattooed region was modeled as a 0.3-mm sublayer embedded within the reticular dermis (centered at 1.4 mm, spanning 1.25–1.55 mm below the skin surface), consistent with common tattoo pigment distributions [27]. Dermis density within the tattooed region was defined as a mass-fraction mixture of native dermal tissue and dry tattoo pigment according to the loading fraction. Mixture densities and corresponding material mass fractions were computed based on the elemental compositions and bulk densities of each tattoo pigment and the reticular dermis. For example, the brown ink (Fe-rich) was assigned to a bulk density of 5.3 g/cm^3^, representative of its metallic oxide, while the reticular dermis density was set to 1.1 g/cm^3^. To study different pigment loadings, mixtures containing 5, 10, 25, 50, 75, and 100 vol% tattoo ink were modeled in a 0.30 mm sublayer. Mixture densities were computed by volume-fraction mixing (see Table 1):

**Table 1:** Tattoo-dermis mixture densities used in the Monte Carlo simulations at different volume fractions.

Pigment loading (vol%)	Dermis fraction (%)	Mixture density (g/cm^3^)		
		Brown	Orange	Black
0 (no ink)	100	1.1	1.1	1.1
5	95	1.3	1.2	1.1
10	90	1.5	1.2	1.2
25	75	2.1	1.4	1.3
50	50	3.2	1.8	1.5
75	25	4.2	2.1	1.6
100	0	5.3	2.4	1.8


$$\rho_{\text{mix}}=v_{\text{ink}}\rho_{\text{ink}}+\left(\text{1}-v_{\text{ink}}\right)\rho_{\text{dermis},}$$

Material composition mass fraction was also calculated accordingly. Corresponding mass fractions used for material composition in TOPAS were obtained using:


$$w_{i}=v_{i}\rho_{i}/\sum_{j}v_{j}\rho_{j}$$

### Monte Carlo simulation

Monte Carlo simulations were performed using TOPAS v3.8.1 (Geant4.10.07.p03) to model electron and photon transport through the multilayer voxelized skin phantom [28–30]. Irradiation conditions included 6 MeV and 18 MeV electron beams and 6 MV and 18 MV photon beams, each with a 1 × 1 cm^2^ field size and an SSD of 100 cm (Fig 1). The 1 × 1 cm^2^ field size was selected to approximate the lateral dimensions of typical tattoo regions. Electromagnetic interactions were modeled using the G4em-Livermore physics list for enhanced low-energy electron transport accuracy. A production cut of 0.001 mm was applied within the tattooed region to resolve micro-scale energy gradients. Each condition was simulated with ≥ 1 × 10^7^ primary particles, yielding statistical uncertainties below 1% within the tattooed region. Dose scoring employed a 3D mesh covering the full 2.4 mm skin, with 0.1 mm binning in z and 2 mm in x–y. DoseToWater (water-equivalent absorbed dose) was recorded in accordance with the standard skin dosimetry conventions. All reported dose values represent mean ±1 standard deviation from Monte Carlo statistics.

All simulations were performed at the University of Chicago Research Computing Center (RCC) Midway3 cluster (partition: caslake). Each simulation utilized 8 CPU cores (Intel Xeon Gold 6346 @ 3.10 GHz, and nodes with 180 GB of system memory. A simulation with 1 × 10^7^ particle histories required approximately 6 minutes for the present geometry and physics settings. Considering the four irradiation modalities investigated in this study, the total runtime per phantom configuration was approximately 2 hours.

### Data analysis

TOPAS outputs were exported as ASCII.csv files containing X, Y, Z, and Dose columns. Analyses were performed in Python using standard scientific libraries (NumPy, Pandas, and Matplotlib/Seaborn). Depth-dose curves and two-dimensional (2D) dose maps were generated to visualize dose perturbations across skin layers and pigment loadings. Depth-dose curves were obtained by averaging dose values laterally at each depth:


$$D_{\text{depth}}(z)={\text{mean}}_{x,y}\left[D(x,y,z)\right]$$

Integrated skin dose was computed by summing dose contributions across all depth bins within the top 2.4 mm. The dose enhancement factor (DEF) was defined as the ratio of tattooed to control skin dose:


$$\text{DEF}=\frac{D_{\text{tattoo}}}{D_{\text{control}}}$$

Two-dimensional (X–Y) dose maps within the scoring region (0–2.4 mm) were generated using a fixed color scale and consistent dose units (Gy) to evaluate lateral dose uniformity across different tattoo loadings and irradiation modalities.

## Results

### Tattooed-skin phantom geometry for simulations

Using the ink compositions defined in Materials and Methods Section, we first established a combined chemical-anatomical framework. Three commercial inks (brown, orange, and black) were selected to span a representative range of pigment compositions from low-Z to high-Z formulations. Elemental contents were extracted from legacy batches reported in the prior survey [4]. Table 2 summarizes the concentrations of key metals in the selected Intenze inks in comparison with EU REACH limits [7]. The brown and orange inks contain the highest levels of regulated species, with Ni, Co, and Pb each exceeding allowable limits. Chromium exceeds the 0.5-ppm threshold in all three inks, including the black ink, indicating a broad prevalence of Cr contamination across pigment types. The brown ink is Fe_2_O_3_-dominant with Fe present at nearly 9 wt% (wet basis) and accompanying impurities spanning sub-ppm to hundreds of ppm, consistent with the compound-based density described in Section 2.1. The orange ink exhibits markedly higher Al, reflecting its Al(OH)_3_ additives. The black ink carried the lowest total metal content, though Cr and Cu remain at or above regulatory thresholds.

**Table 2:** Elemental composition of selected Intenze tattoo inks compared to EU REACH regulatory limits. Multiple toxic metals, including Cr, Ni, Co, and Pb, exceed allowable thresholds in brown and orange inks, with Cr contamination observed across all three formulations. The brown ink exhibits the highest overall metal loading (Fe_2_O_3_-dominant at 88,400 ppm Fe, wet weight) and broadest compositional diversity, representing a conservative upper-bound scenario for dosimetric modeling.

Z-Element	Brown (ppm)^a^	Orange (ppm)^a^	Black (ppm)^a^	Limit (ppm)^b^	REACH Compliance^a^
13-Al	105	1,830	9.36	N/A	–
23-V	11.0	19.3	0.098	N/A	–
24-Cr	147	2.99	1.55	0.5	Exceeds (All)
26-Fe	88,400	0.89	6.42	N/A	–
27-Co	6.44	32.4	0.011	0.5	Exceeds (Brown, Orange)
28-Ni	9.59	13.0	0.070	5	Exceeds (Brown, Orange)
29-Cu	260	<LoQc	5.02	250	Exceeds (Brown)
48-Cd	0.35	0.14	0.013	0.5	Within (All)
56-Ba	19.3	0.29	0.17	500	Within (All)
80-Hg	0.15	0.32	0.011	0.5	Within (All)
82-Pb	8.13	1.51	0.057	0.7	Exceeds (Brown, Orange)

^a^Concentrations converted to ppm from wet-mass data in Ref. [4]. Legacy batches are used to bracket realistic worst-case compositions likely encountered in practice. ^b^REACH thresholds shown where applicable to toxic species; “N/A” where no specific ppm limit applies. ^c^LoQ = limit of quantification.

Among three formulations, the brown ink provides the highest and most compositionally diverse high-Z loading, making it the most conservative boundary condition for evaluating radiological perturbations. Overall, these compositions demonstrate that commercial inks can contain non-trivial amounts of high-Z metals, providing a conservative basis for subsequent dose-interaction modeling.

Fig 1 illustrates the resulting skin-phantom geometry. The embedded tattoo sublayer, modeled using the ink-specific compositions (see Section 2.2 and Table 1), was positioned within the region of steep gradient characteristic of superficial EBRT. This placement enables analysis of both photon- and electron-driven interaction pathways, including photoelectric absorption, forward/backscatter distributions, and modulation of secondary electron fluence. The framework thereby supports systematic comparison of dose perturbations across beam modalities evaluated in this work.

### Dose enhancement by modality and energy

**Fig 2:**
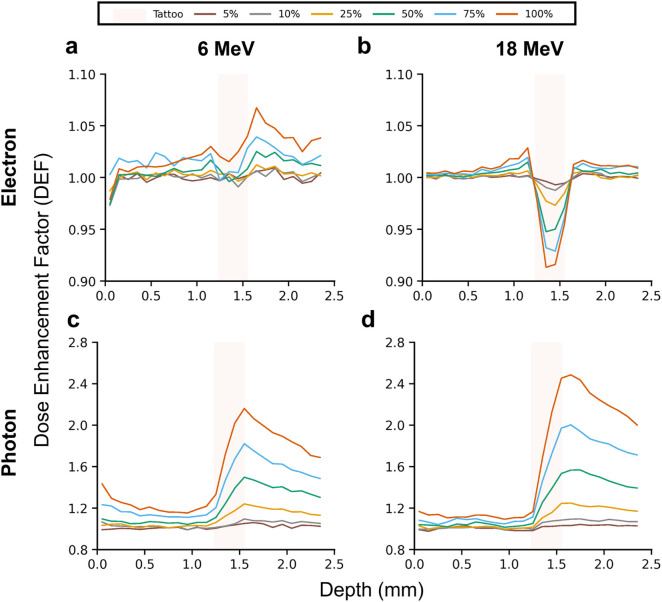
Modality- and energy-dependent dose enhancement factors (DEF) for Fe_2_O_3_-dominant brown tattoo pigment. (a) 6 MeV and (b) 18 MeV electrons, and (c) 6 MV and (d) 18 MV photons. Electron beams produce modest perturbations, with 6 MeV showing enhancement (peak DEF ~ 1.07) while 18 MeV exhibits dose deficits (DEF < 1.0). In contrast, photon beams generate pronounced enhancement (peak DEF ~ 2.2–2.5) driven by high-Z-mediated secondary electron production. Curves shown here for 5%, 10%, 25%, 50%, 75%, and 100% pigment loading.

Dose enhancement was evaluated for the Fe_2_O_3_-dominant brown ink across four clinical energies: two electron (6 and 18 MeV) and two photon energies (6 and 18 MV). Fig 2 shows all pigment loadings (5–100%) exhibited similar qualitative ordering of effects, with higher pigment fractions producing larger perturbations while preserving the overall trend, demonstrating a concentration-dependent response.

Under electron irradiation, 6 MeV beams produced shallow but distinct enhancement localized to the reticular dermis, with peak DEF of ~1.07 at 100% pigment loading (Fig 2a). The dose increase occurred within and slightly above the pigment layer, with the tattoo-layer mean dose rising by ~2.5%. However, this enhancement was highly energy dependent. At 18 MeV, the behavior reversed; rather than showing dose enhancement, the brown ink produced localized dose deficits (DEF < 1.0), with peak DEF reduced to ~1.03 and mean DEF approximately 1.00 (Fig 2b).

In sharp contrast, photon irradiation yielded substantially larger perturbations than electron beams across all energies. The 6 MV photons generated peak DEF of ~2.16 (Fig 2c), while 18 MV photons reached ~2.49 (Fig 2d). Corresponding mean DEF values were 53% and 62% at 6 and 18 MV, respectively, under the theoretical 100% pigment-loading condition, representing an upper-bound scenario. These enhancements arise from increased secondary-electron production in the high-Z pigment. For 6 MV photons, elevated electron density in Fe-rich pigment increases the probability of Compton interactions, generating more high-energy electrons in the surrounding dermis. At 18 MV, additional contributions from pair production become significant, further amplifying dose deposition. Across 5–100% pigment loadings, the curves preserved the same qualitative shape. At lower loadings (5 vol%), the mean dose deviation remained within 5%, while localized peak perturbations slightly exceeded this threshold. Larger deviations above 5% became more apparent at higher pigment fractions (>10 vol%).

### Depth-dose profiles under electron and photon irradiation

**Fig 3:**
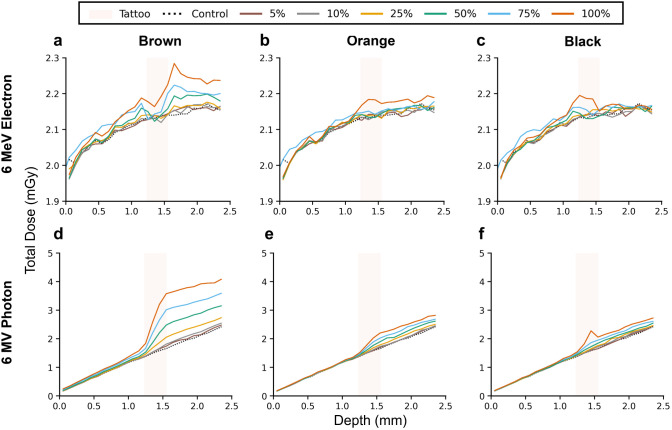
Composition-dependent depth-dose profiles for three inks under 6 MeV electron and 6 MV photon irradiation. (a, d) brown (Fe_2_O_3_-rich), (b, e) orange (Al-containing), and (c, f) black (carbon-based). Under electron irradiation, all pigments show shallow, localized perturbations with total skin-integrated dose differing by <2.5% from control. Under photon irradiation, dose perturbations follow the trend brown > orange > black, reflecting each ink’s high-Z metal content. Pigment loadings: 0%, 5%, 10%, 25%, 50%, 75%, and 100%.

Having established the modality dependence using the brown ink, we next examined how these trends across both electron and photon beams and across the three ink formulations. Fig 3 compiles raw total dose-depth profiles for brown, orange, and black inks under 6 MeV electron and 6 MV photon irradiation at pigment loadings from 0% to 100%.

Under 6 MeV electron irradiation (Fig 3a–c), all three pigments exhibited shallow, localized perturbations that remained confined to the reticular dermis. Consistent with Section 3.2, brown ink showed the localized increase of ~ 6–7% at 100% pigment loading (Fig 3a). The other two inks exhibited smaller localized enhancements of ~ 2–3% at full loadings, reflecting their lower high-Z content. Despite these localized features, the total dose delivered to the skin slab (0–2.4 mm) differed by < 2.5% relative to the no-ink control for all three pigments and concentrations (Fig 2e), and the corresponding DEF remained within 1.00–1.03.

Under 6 MV photon irradiation (Fig 3d–f), the dose-depth profiles exhibited more distinct pigment- and concentration-dependent perturbations due to the large dose perturbations shown in Fig 2c. Color-dependent trends persisted across the entire superficial region: brown > orange > black, consistent with the 6 MeV electron results mentioned above. These findings show that tattoo pigments introduce localized depth-dependent variations whose magnitude correlates with the high-Z fraction of the pigment.

### Spatial dose perturbations

**Fig 4:**
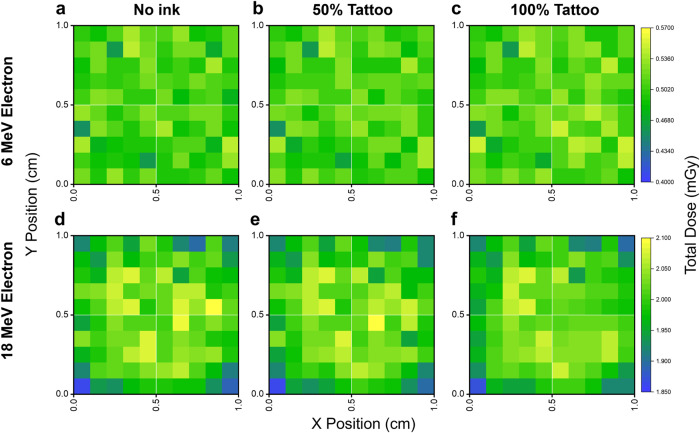
Electron-beam spatial dose distributions for a multi-color tattoo geometry (1 × 1 cm^2^, four 0.5 × 0.5 cm^2^ quadrants). No ink (top-left), black (top-right), orange (bottom-left), and brown (bottom-right). Dose maps shown for 6 MeV(a–c) and 18 MeV (d–f) electron at 0%, 50%, and 100% pigment loading. Electron irradiation produces laterally uniform dose distributions.

Furthermore, to determine whether tattoo pigments introduce lateral dose heterogeneity, 2D dose maps were generated for both the electron and photon irradiation conditions using a four-quadrant, multi-color tattoo configuration. Under 6 and 18 MeV electron irradiation, the dose maps in Fig 4 show largely uniform lateral dose distributions across all pigment loadings. While local pixel-scale fluctuations are visible, particularly at 100% pigment loading (Fig 4c, f), these variations do not form coherent quadrant-wide patterns. Notably, although the brown-pigment quadrant (bottom right) exhibits slightly higher frequency of locally elevated pixels at full loading, this effect remains spatially diffuse and does not produce a systematic lateral dose shift across the tattoo plane.

**Fig 5:**
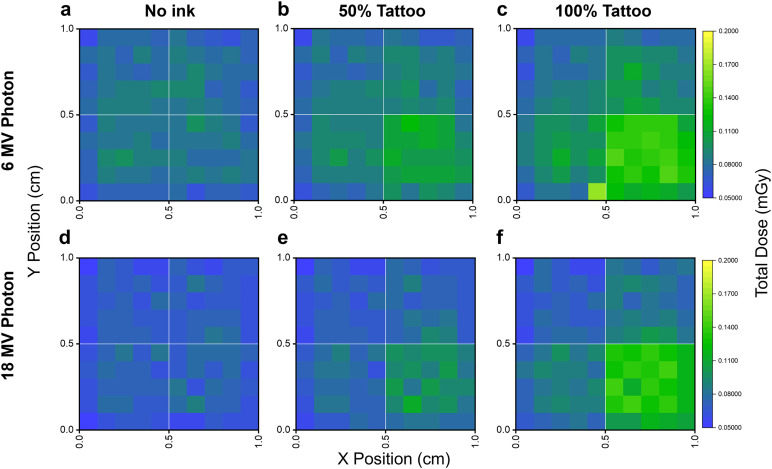
Photon-beam spatial dose distributions for a multi-color tattoo geometry (1 × 1 cm^2^, four 0.5 × 0.5 cm^2^ quadrants). No ink (top-left), black (top-right), orange (bottom-left), and brown (bottom-right). Dose maps shown for 6 MV (a–c) and 18 MV (d–f) photon at 0%, 50%, and 100% pigment loading. Photon irradiation shows clear geometry-correlated enhancement patterns, but perturbations remain spatially confined to tattooed regions without lateral propagation.

Under 6 and 18 MV photon irradiation, the dose maps in Fig 5 reveal clear and structured geometry-correlated dose patterns. As pigment loading increases, tattoo-containing quadrants show consistent and spatially coherent dose enhancement relative to the no-ink region (top left), with the effect most pronounced at 100% loading (Fig 5c, f). These patterns directly reflect the underlying pigment distribution, indicating a fundamentally different lateral sensitivity compared to electron irradiation. Together, these results demonstrate that while electron irradiation may exhibit minor local fluctuations at high pigment loadings, clinically relevant lateral dose modulation emerges predominantly under photon irradiation, where tattoo composition and spatial arrangement directly imprint onto the dose distribution.

## Discussion

The three tattoo inks examined in this work exhibited clear composition-dependent dose perturbations that reflected their differing metal contents (Table 2). The Fe_2_O_3_-dominant brown ink, containing higher levels of high-Z elements made up of mostly Fe and other metals like Cr, Ni, Co, *etc*., produced the largest dose deviations. The orange (Al-containing) and the black ink (C-based, low in metal content) generated comparatively smaller perturbations (Fig 3). This trend follows established relationships between effective atomic number, pigment density, and transport of secondary electrons in heterogeneous skin environments [31]. Spatial dose maps in Figs 4 and 5 revealed that pigment composition did not introduce lateral dose non-uniformity for either modality. Even at 100% pigment loadings of multi-color tattoo geometries (Fig 4c, f and Fig 5c, f), consisting of brown, orange, black, and no-ink, gave no coherent brightening, attenuation, or spatial imprinting across the irradiated plane. These results confirm that tattoo pigments perturb dose primarily along the depth axis, while lateral transport remains uniform across the field.

Under electron irradiation, the brown ink generated depth-localized enhancements of approximately 7% at 6 MeV (Fig 2a and 3a). In contrast, at 18 MeV, the energy-dependent transition to dose deficits (DEF < 1.0, Fig 2b) demonstrates that higher-energy electrons can reduce local dose in tattooed regions, an outcome opposite to the enhancement observed with lower-energy electron (6 MeV) and all photon conditions (6, 18 MV).

This counterintuitive transition reflects the competing mechanisms in high-Z heterogeneities [31,32]. At 6 MeV, backscatter and secondary electron production from Fe_2_O_3_ dominate, leading to net dose enhancement confined to the vicinity of the pigment layer. At 18 MeV, electrons possess sufficient energy to penetrate through the pigment layer with limited range perturbation. Although scattering probability remains elevated in high-Z material, the longer mean free path and more forward-peaked angular distribution result in enhanced lateral deflection rather than localized energy deposition. Consequently, a fraction of primary electrons is redistributed away from the tattoo region, creating a dose deficit in the tissue immediately beneath the pigment layer. Because electron-induced perturbations remain confined in depth and do not extend laterally, the depth-integrated superficial dose outside the tattoo region was minimally affected in both electron energies, regardless of whether local dose increased (6 MeV) or decreased (18 MeV).

In contrast, photon beams yielded substantially larger and more depth-extended perturbations than electron beams. Peak DEF values exceeded a factor of two to three, and mean DEF values increased by 53% (6 MV) and 62% (18 MV) relative to no-ink control under the theoretical 100% pigment-loading condition. To ensure patient safety, these values represent a clinically conservative upper-bound scenario designed to evaluate potential risks under extreme conditions. Therefore, they should be interpreted as parametric limits that may overestimate the dose perturbation magnitude for typical tattoos, where pigments are dispersed within dermal tissue at lower effective volume fractions. Based on the pigment mass loadings reported by Engel et al. (0.6–9.4 mg/cm^2^ for a red azo dye as model tattooed skin) and the tattoo layer thickness used in the present model (0.3 mm) [33], these values correspond to approximate pigment volume fractions up to 25% assuming typical azo organic densities.

The enhanced doses in the presence of high-Z pigment are governed by Compton scattering and, at higher energies, pair production [34]. For 6 MV photons, enhanced electron density with Fe-rich brown pigment increases the probability of Compton interactions, leading to elevated number of high-energy electrons in the surrounding dermis. At 18 MV, additional contributions from pair production became significant. Owing to the Z dependence, pair production within the Fe_2_O_3_-dominant pigment generates energetic electron-positron pairs whose extended ranges and subsequent annihilation processes further increase secondary particle transport and dose spread. This combined increase in secondary electrons explains the systematically higher DEF observed for 18 MV relative to 6 MV photons. Across 5–100% pigment loadings, the depth-dose curves in Fig 3c, d preserved the same qualitative shape, confirming high-Z-mediated photon interaction mechanisms dominate the observed dose enhancement.

Although the photon-induced perturbations remain confined to the dermis, the depth range of maximal enhancement corresponds predominantly to the reticular dermis rather than the superficial papillary layer. This compartment contains dense vascular networks, adnexal structures such as sweat glands and hair follicles, and exhibits a higher capacity for inflammatory signaling. As a result, localized dose perturbations in the reticular dermis are likely to be relevant to radiation dermatitis. Such effects may be particularly consequential in treatment settings where superficial dose drives toxicity [35], including breast irradiation, head-and-neck radiotherapy, and other skin dose-limited cases.

Notably, current treatment-planning–system (TPS) algorithms do not explicitly account for tattoo-specific high-Z pigments or their depth-dependent transport behavior [16], suggesting that these localized dose enhancements can be systematically under-represented when tattoos lie within or adjacent to the treatment field. Although volumetric tattoo-specific data are beyond the scope of this work, the localized dose increases observed within the dermal layers imply that sufficiently large or pigment-dense tattooed areas could experience non-negligible increases in dose-volume metrics such as V_30_/V_40_/V_50_ Gy. In clinical contexts where hypofractionation or stereotactic body radiation therapy (SBRT) is used, high-dose per fraction may amplify both acute and late reactions [36], further supporting caution when photon beams traverse above pigment-dense tattoos. While comprehensive risk stratification will require future studies, the present findings highlight tattoo pigments as a modest but previously unmodeled source of uncertainty in skin-dose estimation. The modality-dependent behavior reflects fundamental differences in energy deposition mechanisms: electrons deposit dose directly and are rapidly attenuated, limiting perturbations to the immediate vicinity of the high-Z inclusions; photons, by contrast, generate secondary electrons through Compton and pair-production interactions that propagate into adjacent tissue layers [37]. These findings suggest that tattooed regions may warrant additional consideration during photon treatments involving shallow targets, skin-dose constraints, or bolus use [15]. Further characterization of modern pigment formulations and *in-vivo* validation will be essential to define clinical protocols.

From a regulatory and translational perspective, radiological behavior represents only one dimension of tattoo-pigment safety. Chemical toxicity, impurity control, and microbiological contamination remain active concerns within U.S. and international regulatory frameworks. The recent implementation of the Modernization of Cosmetics Regulation Act by FDA (MoCRA, 2022) reflects ongoing efforts to strengthen oversight of pigment quality, particularly in relation to manufacturing hygiene and microbiological safety [38]. Of additional relevance is the unregulated commercial availability of tattoo inks through consumer retail channels, where products containing non-compliant metal concentrations remain accessible to the general public [8,9], a population of which a substantial proportion will require radiotherapy at some point of their lifetime. Although the inorganic components in tattoo inks are present at lower concentrations than engineered metal, metal oxides and chalcogenides, the underlying principles governing their interactions with ionizing radiation remain similar. Under megavoltage photon irradiation, high-Z inorganic materials such as Fe_2_O_3_ or Au exhibit distinct scattering and charged-particle transport characteristics relative to surrounding tissues. In particular, Compton-mediated secondary electron generation combined with altered electron transport and stopping behavior in dense pigment regions may contribute to localized variations in energy deposition at pigment-tissue interfaces. Such localized dose amplification has been linked, in other material-biological systems, to enhance redox chemistry and reactive oxygen species formation [39–41]. These considerations underscore the need for a multidisciplinary safety framework that integrates radiological, chemical, and biological evaluation of tattoo pigments.

This study has several limitations. The findings are derived from Monte Carlo simulations using idealized multilayer skin phantoms, which necessarily simplify patient-specific anatomical variability. From a chemical standpoint, the pigment compositions modeled here represent approximations of reported formulations, and deviations in actual ink composition, aggregation state, or pigment-protein interactions could alter the magnitude of dose perturbation. Moreover, the pigment loading and effective density used in the simulations represent simplified sensitivity scenarios and may overestimate the effective pigment volume fraction present in typical tattooed skin. Accordingly, the model should be interpreted as a representation of pigment accumulation in vivo. In addition, this work focused on photon and electron beams; interactions involving particle beams such as protons or carbon ions, of increasing clinical relevance, remain uncharacterized.

Furthermore, the beam sources were modeled as monoenergetic with Gaussian energy spread rather than using phase-space files from commissioned linear accelerator head simulations. While this simplified approach is appropriate for systematic comparison across energies and pigment compositions, it does not account for spectral components such as low-energy scatter photons, contaminant electrons, or head-leakage radiation that may be present in clinical beams. Future work incorporating realistic phase-space distributions, e.g., IAEA phase-space files for generic clinical linear accelerators [42], together with patient-specific tattoo geometry and composition, and experimental validation, will help refine the magnitude of predicted dose perturbations and establish clinical relevance for tattooed patients undergoing radiation therapy.

## Conclusion

Tattoo pigments containing iron oxides and trace metals induced modality-dependent, depth-localized perturbations, with photon beams producing the largest dose enhancement effects under high-density simulation conditions. While electron beams generated modest and clinically limited changes, photon irradiation created pronounced dosimetric hotspots driven by high-Z-medicated secondary electron production. These localized enhancements do not necessarily translate to large field-integrated dose changes, yet their magnitude in the dermis suggests that tattoos may represent an under-recognized factor when treating shallow targets or managing skin-dose constraints. A more comprehensive understanding of pigment chemistry and patient-specific geometry, coupled with experimental in-vivo or ex-vivo validation, will be critical for developing evidence-based guidance and ensuring patient safety, especially in photon-based EBRT.
